# Prevalence of the metabolic syndrome and its components in Northwest Russia: the Arkhangelsk study

**DOI:** 10.1186/1471-2458-10-23

**Published:** 2010-01-19

**Authors:** Oleg Sidorenkov, Odd Nilssen, Tormod Brenn, Sergey Martiushov, Vadim L Arkhipovsky, Andrej M Grjibovski

**Affiliations:** 1 Institute of Community Medicine, University of Tromsø, postbox 9037 Tromsø, Norway; 2 Department of Internal Medicine-II, Northern State Medical University, Troitsky Ave, 51, Arkhangelsk 163001, Russia; 3 Semashko Clinic, Arkhangelsk, Russia; 4 Norwegian Institute of Public Health, Postbox 4404 Nydalen, 0403 Oslo, Norway; 5 International School of Public Health, Northern State Medical University, Troitsky Ave, 51, Arkhangelsk 163001, Russia

## Abstract

**Background:**

The metabolic syndrome (MetS) is a cluster of risk factors associated with morbidity from cardiovascular disease (CVD) and associated mortality. Russia has one of the highest CVD mortality rates in the world. However, the prevalence of MetS in Russia remains largely unknown. The aim of this study is to estimate the prevalence of MetS and its components in an urban Russian setting.

**Methods:**

Altogether, 3705 Russian adults aged 18-90 years were enrolled in a cross-sectional study in Arkhangelsk (Northwest Russia). All subjects completed a questionnaire and underwent a physical examination. Blood samples were taken and analyzed in TromsØ, Norway. Three separate modified definitions of MetS were used, namely, the National Education Cholesterol Education Program Adult Treatment Panel III (NCEP), the American Heart Association/National Heart, Lung and Blood Institute (AHA/NHLBI) and the International Diabetes Federation (IDF). To ensure comparability of the findings, the prevalence data were standardized using world and European standard populations and Russian population.

**Results:**

The age-standardized (Segi's world standard population) prevalence rates of the MetS among women were 19.8% (95% CI: 18.1-21.5), 20.6% (95% CI: 18.9-22.3) and 23.1% (95% CI: 21.3-24.9) by the NCEP, AHA/NHLBI and IDF criteria, respectively. The corresponding rates for men were 11.5% (95% CI: 10.1-12.9), 13.7% (95% CI: 12.2-15.2) and 11.0% (95% CI: 9.7-12.4). Among subjects with MetS, central obesity was more common among women, while elevated triglycerides and blood glucose were more common among men. Almost perfect agreement was found between the NCEP and AHA/NHLBI criteria (κ = 0.94). There was less agreement between the used definitions of MetS in men than in women.

**Conclusions:**

While the prevalence of MetS among Russian women is comparable to the data for Europe and the U.S., the prevalence among Russian men is considerably lower than among their European and North-American counterparts. Our results suggest that MetS is unlikely to be a major contributor to the high cardiovascular mortality among Russian men. Further studies of MetS determinants and associated cardiovascular risk are needed for a better understanding of the mechanisms leading to the exceptionally high cardiovascular mortality in Russia.

## Background

MetS is an unfavourable cluster of factors that increases the risk of CVD and type-2 diabetes [1-3]. MetS is associated with more than 50% increased risk of cardiovascular mortality and an almost 30% enhanced risk of mortality from all causes [4-6]. It is a considerable public health issue in both developed and developing countries. In general, its prevalence in Europe and among Americans of European descent varies between 20% and 30%, with approximately equal distribution by gender [7-11]. Although genetic predisposition has been suggested as an important determinant of MetS [12], genetic factors alone cannot explain the recent increase in prevalence in both Europe and the U.S.

Internationally, there is no uniform accepted definition of MetS. Altogether, six sets of diagnostic criteria have been proposed by different expert groups. Despite considerable similarity among the definitions, the prevalence of the MetS in the same population may vary dramatically depending on the specific diagnostic criteria considered [13]. This complicates international comparisons and may challenge estimates of the global burden of the syndrome.

While cardiovascular mortality in Western Europe and the U.S. has decreased during recent decades, the opposite trend has been observed in Russia where it has increased from 412 per 100,000 in 1970 to 927 per 100,000 in 2003. Mortality from cardiovascular diseases in Russia is currently the highest in Europe. In 2003, CVDs accounted for about 56% of all deaths [14]. Coronary heart disease and cerebrovascular diseases alone, respectively, constituted 26 % and 20% of the total mortality. The highest increase in CVD has occurred among 30-60 year-olds, particularly among men [15].

Given that MetS is a strong predictor of cardiovascular mortality and morbidity [4-6], one may suspect a high prevalence of this syndrome in contemporary Russia. Few studies have described the prevalence of dyslipidemia, hypertension and obesity among Russians [16,17]. The actual rates reported were either comparable to or lower than those in Europe [18]. These studies, however, focused only on the distribution of major cardiovascular risk factors. To the best of our knowledge, no large Russian population-based studies on cluster of the major cardiovascular risk factors, such as the MetS, have been published.

The aim of the present study is to estimate the prevalence of MetS and its components in an urban Russian setting using several international definitions and reference populations to ensure comparability of the findings.

## Methods

### Sample characteristics

The survey was conducted in 2000 in Arkhangelsk, the capital of the Arkhangelsk Region of Russia. The population of Arkhangelsk prior to the study's initiation was approximately 170,000 men and 197,000 women. The city is ethnically homogenous: 95% of inhabitants are registered as Russians and most of the reminder are ethnically and culturally close to Russians (e.g. 2% of the population are registered as Ukrainians and 1% as Byelorussians).

No population register for medical research exists in Arkhangelsk. Primary health care departments provide medical services to the general population within the regional general health and occupational health network. People are registered at polyclinics according to their home address and/or place of work. All study participants when registered at the same out-patient clinic in Arkhangelsk. Of those who were invited, only 40 persons (1.1 %) refused to participate with "lack of time" as the primary reason given. Individuals coming for their annual medical check-up at the out-patient clinic were recruited consecutively to avoid the "healthy volunteer effect". Workers and students were similarly invited either through the obligatory annual medical examination or through their places of work or study. Pensioners were recruited through the clinic's register. About 90% of males and 70% of females were recruited through an annual medical examination consisting mainly of working people but also students, pensioners and unemployed individuals. Other subjects were invited to the study. Students constituted approximately 12%, pensioners 19%, unemployed 3% and working subjects 66 % of the study population.

Altogether, 1968 men and 1737 women aged 18-90 years participated in the study. It involved a physical examination, completion of a comprehensive questionnaire and donating blood for tests. Data were collected by trained medical personnel. The study was approved by the Regional Ethics Committee, TromsØ, Norway. Verbal informed consent was obtained from all participants.

### Data collection

Anthropometric measurements included weight, height, waist and hip circumference. Body mass index (BMI) and waist-to-hip ratio (WHR) were calculated. Blood pressure and heart rate were measured (upper right arm) in a sitting position three times at two minute intervals using a semiautomatic electronic device (DINAMAP-R, Criticon, Tampa, Florida). Averages of the second and third systolic and diastolic blood pressure readings were used in the analysis. In addition, all subjects completed a six-page questionnaire on socio-demographic characteristics, medicines used (including regular intake of antihypertensive and anti-diabetic medications), smoking habit, alcohol consumption, diet and level of physical activity during leisure time and at work. History of cardiovascular diseases and diabetes were assessed by additional questions preceded by "do you now have or have you ever had" angina pectoris (AP), myocardial infarction (MI), stroke or diabetes mellitus (DM). Only basic socio-demographic data and self-reported diseases are presented for descriptive purposes in this paper. Age was categorized into five groups: 18-29, 30-39, 40-49, 50-59 and 60+ years. Education was classified as secondary or lower, vocational, incomplete higher, or higher. Income level was very difficult to determine during the year 2000 owing to high inflation and a collapsing economy, with about 30% of the population (official data) having incomes below the survival minimum[19]. Consequently, we used self-reported occupational status data as a surrogate measure of income. Assigned income levels were based on the official year-2000 average salary levels recorded for different sectors of the economy [20], and were categorized as very low, low, medium or high. The income of groups for whom there were no official salary data (for example, students, the unemployed and housewives) was classified as unknown. Cigarette smoking was categorized as "yes" (occasional or daily smokers) or "no" (non-smokers or ex-smokers). Data on frequency of alcohol consumption were obtained by asking "How often do you drink alcoholic beverages?". More details about the study, recruitment details, sample and data collection protocols are presented elsewhere [21,22].

### Laboratory measurements

Venous blood samples were drawn in the morning and centrifuged within 15-25 minutes in the laboratory at the study site in Arkhangelsk. Because subjects generally fast in preparation for these annual medical check-ups, since screening for diabetes and impaired glucose tolerance is part of the examination, we assume that most of the study participants indeed fasted. Nevertheless, none of them was directly asked by the study team to fast prior to the medical examination. The serum samples were stored at -20°C and then transported frozen to Norway where they were kept at -80°C pending analysis. Total serum cholesterol (TC), triglycerides (TG), high-density lipoprotein cholesterol (HDL-C), serum glucose (SG) and glycosylated hemoglobin (HBA1c) were measured. All laboratory analyses were carried out at the Department of Clinical Chemistry, University Hospital of Northern Norway (UNN) in Tromsø.

Enzymatic colorimetric tests were used to measure TC (cholesterol esterase, cholesterol oxidase) and TG (lipoprotein lipase, glycerokinase, and glycerophosphate oxidase). HDL-C was measured by a homogenous enzymatic colorimetric test (PEG cholesterol esterase, and PEG peroxidase). The coefficients of variation (CV) were, respectively, 5%, 2% and 3% for the TC, TG and HDL-C determinations. All biochemical analyses of serum lipids were performed using a Hitachi 737 analyzer. SG was measured by the hexokinase method using a Hitachi 917 analyzer (CV = 2%). HBA1c concentration was determined using the Bio-Rad Variant II HPLC system with reagents from Bio-Rad Laboratories (Inc., Hercules, CA 94547, USA), with CV <5%. The laboratory routinely participates in formal quality assurance exercises.

### Definition of the metabolic syndrome

MetS was defined according to the National Cholesterol Education Program Adult Treatment Panel III (NCEP) [23], the American Heart Association/National Heart, Lung and Blood Institute (AHA/NHLBI) version [24] and the International Diabetes Federation (IDF) [25].

### Statistical analysis

The prevalence estimates were standardized by age using Segi's world standard population, European standard population and Russian population (based on data from the National Census in 2002) [26]. The following age-strata were used: 20-29, 30-39, 40-49, 50-59 and 60+ years. Ninety-five percent confidence intervals (CI) were calculated for all prevalence estimates. Gender differences in socio-demographic and some life-style characteristics were compared using Pearson's chi-squared tests and unpaired t-tests for categorical and numerical data, respectively. To identify sex-specific cut-offs for waist circumference values corresponding to BMIs of ≥25 kg/m^2 ^and ≥30 kg/m^2^, a receiver operating characteristic (ROC) analysis was carried out. Agreement between different diagnostic criteria for MetS was assessed by Cohen's kappa statistic. All analyses were performed using SPSS version 14 (SPSS Inc, Chicago, IL).

## Results

### Description of the study sample

Among the 3705 study participants, 150 (4.0%) had missing data on one or more variables and were therefore excluded. The final study group included 1918 men and 1637 women, corresponding to 95% of all those invited.

The women were slightly older and were better educated, but had much lower income and a higher prevalence of self-reported diseases than men. The men smoked more and took alcohol more frequently (Table 1).

**Table 1:** Baseline characteristics of the sample

Sample characteristic		Men		Women	P^1^
	N	%	N	%	
*Age, years*					0.002
18-29	515	26.8	347	21.2	
30-39	352	18.4	303	18.5	
40-49	441	23.0	400	24.4	
50-59	298	15.5	290	17.7	
60+	312	16.3	297	18.1	
*Marital status*					<0.001
Single	409	21.3	278	17.0	
Married	1276	66.5	878	53.6	
Divorced	82	4.3	182	11.1	
Widow(er)	60	3.1	219	13.4	
Cohabiting	91	4.7	80	4.9	
*Education*					<0.001
Secondary	435	22.7	426	26.0	
Vocational	1083	56.5	669	40.9	
Incomplete higher	87	4.5	105	6.4	
Higher	303	16.3	437	26.7	
*Income*					<0.001
Very low	283	14.8	379	23.2	
Low	136	7.1	740	45.2	
Medium	144	7.5	189	11.5	
High	1058	55.2	34	2.1	
Unknown	297	15.5	295	18.0	
Frequency of alcohol intake, %					<0.001
Never	230	12.0	445	27.2	
Once a month or less	434	22.6	542	33.1	
2-4 times a month	979	51.0	571	34.9	
2 times a week or more	275	14.3	79	4.8	
Current smoking, %	1085	56.6	348	21.3	<0.001
*Self-reported diseases*					
Diabetes mellitus	28	1.5	48	2.9	0.002
Coronary heart disease	176	9.2	195	11.9	0.008
Stroke	9	0.5	30	1.8	<0.001

Total	1918	100.0	1637	100.0	

^1 ^Calculated by Pearson's chi-squared test

^2 ^One Alcohol Unit (AU) is equivalent to 13.8 grams of pure ethanol

**Figure 1:**
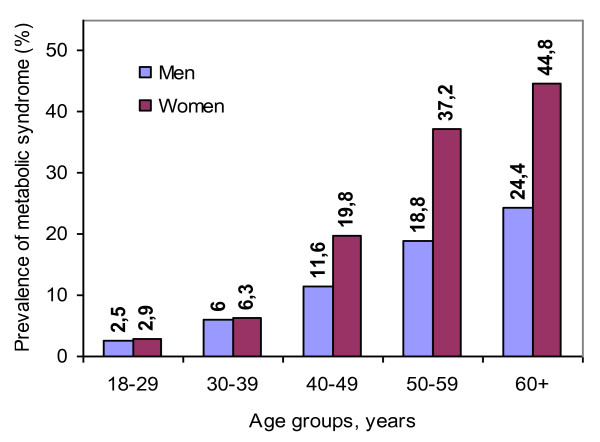
**Sex-specific prevalence of the metabolic syndrome across age-groups according to NCEP definition**.

The prevalence of abnormally high components of MetS as well as BMI and WHR increased with age in both genders (Table 2), as did the overall prevalence of MetS (Figure 1). This increase was more pronounced among women. The prevalence of MetS was similar in men and women in the youngest age group (2.5% vs. 2.9%), but was almost twice as high in women as compared to men in the oldest age group (44.8% vs. 24.4%).

**Table 2:** Proportion of abnormal values (%)^1 ^for the components of the metabolic syndrome as well as body mass index (BMI) and waist to hip ratio (WHR) by age group and gender.

					Age-groups							
	
	18-29 years		30-39 years		40-49 years		50-59 years		60 and over		Total	
	
	%	95 % CI	%	95 % CI	%	95 % CI	%	95 % CI	%	95 % CI	%	95 % CI
Women												
TG	6.1	3.9-9.2	9.9	6.9-14.0	21.3	17.4-25.7	29.7	24.5-35.3	37.4	31.9-43.2	20.3	18.4-22.4
HDL-C	36.3	31.3-41.6	36.3	30.9-42.0	38.0	33.3-43.0	49.3	43.4-55.2	54.5	48.7-60.3	42.3	39.9-44.8
DBP	0.3	0.02-1.9	5.6	3.4-9.0	17.3	13.8-21.4	32.8	27.5-38.5	43.4	37.8-49.3	19.0	17.1-21.0
SBP	7.5	5.0-10.9	13.9	10.3-18.4	35.8	31.1-40.7	62.1	56.2-67.6	75.8	70.4-80.4	37.6	35.3-40.0
Glucose	0.6	0.1-2.3	2.6	1.2-5.3	4.3	2.6-6.9	10.7	7.5-15.0	16.5	12.6-21.3	6.5	5.4-7.9
HBA1c	0.3	0.02-1.9	0.3	0.02-2.1	1.8	0.8-3.7	8.3	5.5-12.2	12.5	9.0-16.9	4.3	3.4-5.4
WC	4.3	2.5-7.2	14.5	10.9-19.1	32.5	28.0-37.4	47.6	41.7-53.5	49.8	44.0-55.7	29.0	26.8-31.3
BMI	3.5	1.9-6.1	13.2	9.7-17.7	21.5	17.6-25.9	35.9	30.4-41.7	33.3	28.1-39.1	20.8	18.9-22.9
WHR	3.2	1.7-5.8	7.3	4.7-10.9	18.8	15.1-23.0	31.0	25.8-36.8	33.3	28.1-39.1	18.1	16.3-20.1
Men												
TG	12.0	9.5-15.1	21.3	17.2-26.0	25.4	21.5-29.8	32.2	27.0-37.9	28.2	23.4-33.6	22.6	20.7-24.5
HDL-C	27.2	23.4-31.3	17.1	13.4-21.5	24.5	20.6-28.8	32.9	27.6-38.6	38.1	32.8-43.8	27.4	25.4-29.4
DBP	1.4	0.6-2.9	17.6	13.9-22.1	33.6	29.2-38.2	39.3	33.7-45.1	42.9	37.4-48.7	24.4	22.5-26.4
SBP	22.3	18.9-26.2	39.2	34.1-44.5	52.8	48.1-57.6	68.5	62.8-73.6	78.5	73.5-82.9	48.8	46.5-51.0
Glucose	0.8	0.3-2.1	1.7	0.7-3.9	6.1	4.2-8.9	12.1	8.7-16.5	20.8	16.6-25.9	7.2	6.1-8.5
HBA1c	0.2	0.01-1.2	0	0-1.4	1.1	0.4-2.8	4.4	2.4-7.5	12.5	9.1-16.8	3.0	2.3-3.9
WC	0.8	0.3-2.1	4.3	2.5-7.1	7.5	5.3-10.5	12.1	8.7-16.5	10.6	7.5-14.7	6.3	5.3-7.5
BMI	3.3	2.0-5.3	9.1	6.4-12.7	14.7	11.6-18.5	20.1	15.8-25.2	15.4	11.7-20.0	11.6	10.2-13.1
WHR	4.9	3.2-7.2	25.0	20.6-29.9	30.2	26.0-34.7	37.9	32.4-43.7	46.8	41.2-52.5	26.3	24.4-28.4

^1 ^Proportions of abnormal values (%) with 95 % CI. BMI, body mass index in kg/m^2 ^>30; TG, triglycerides ≥1,7 mmol/l; HDL-C, high-density lipoprotein cholesterol <1,29 mmol/l (women) and <1,04 mmol/l (men); WC, waist circumference ≥88 cm (women) and ≥102 cm (men); DBP, diastolic blood pressure ≥ 85 mmHg; SBP, systolic blood pressure ≥ 130 mmHg; Glucose, serum glucose ≥ 6,1 mmol/l or self-reported DM, or Rt for hyperglycemia; HBA1c, glycosylated haemoglobin ≥ 6,1% or self-reported DM, or Rt for hyperglycemia; WHR, waist-to-hip ratio > 0,85 (women) and >0,9 (men).

The prevalence of obesity (Table 2) varied strikingly depending on the definition employed (WC, BMI or WHR). We performed a ROC analysis to evaluate the applicability of the given WC cut-offs in our study sample. The WC cut-off ≥94 cm identified men with BMI ≥25 kg/m^2 ^with sensitivity (Se) of 0.35 and specificity (Sp) of 0.99. The WC cut-off ≥102 cm identified men having BMI ≥30 kg/m^2 ^with a Se of 0.41 and Sp of 0.99. In men of our study setting, the BMI cut-offs of ≥25 kg/m^2 ^and ≥30 kg/m^2 ^corresponded best to WC of ≥84 cm (Se, 0.82; Sp, 0.85) and 92 cm (Se, 0.88; Sp, 0.86), respectively. The standard WC cut-offs of ≥80 cm and ≥88 cm applied in women, which corresponded respectively to BMIs of ≥25 kg/m^2 ^and ≥30 kg/m^2^, were originally characterized by good test properties (respectively, Se of 0.79 and 0.87, and Sp of 0.91 and 0.88).

### Prevalence of the metabolic syndrome

The overall prevalence of the MetS varied with the definition and reference population used (Table 3). Standardization by Segi's world population gave consistently lower prevalence estimates than standardization by the Russian population. The estimates standardized by the European standard population were almost identical to the latter and are therefore not presented. There was almost perfect agreement between the estimates of MetS in both men and women using the NCEP and AHA/NHLBI diagnostic criteria (Table 4). There was less agreement when the NCEP and IDF criteria were compared, especially among men. A comparable disparity was observed between the AHA/NHLBI and IDF estimates.

**Table 3:** Age-standardized^1 ^prevalence of the metabolic syndrome according to the AHA/NHLBI, NCEP and IDF definitions

	Prevalence, % (95% CI)					
	
	AHA/NHLBI		NCEP		IDF	
	
	World	Russian	World	Russian	World	Russian
Men	13.7 (12.2-15.2)	15.3 (13.6-17.0)	11.5 (10.1-12.9)	12.9 (11.3-14.5)	11.0 (9.7-12.4)	12.3 (10.7-13.8)
Women	20.6 (18.9-22.3)	23.4 (21.5-25.4)	19.8 (18.1-21.5)	22.5 (20.6-24.5)	23.1 (21.3-24.9)	26.0 (24.0-28.0)
Total	17.0 (15.9-18.2)	19.3 (18.0-20.1)	15.5 (11.4-16.6)	17.6 (16.3-18.8)	16.8 (15.7-18.0)	18.9 (17.6-20.2)

^1 ^Standardized to the Segi's World standard population and the Russian population in 2002 by the following age-strata 20-29, 30-39, 40-49, 50-59, 60+ years.

**Table 4:** Kappa statistics (κ) with standard errors (SE) for the agreement between the prevalence of metabolic syndrome estimates obtained by three different diagnostic criteria

Agreement between the diagnostic criteria:	κ (SE)		
	
	Men (N = 1918)	Women (N = 1637)	Total (N = 3555)
NCEP and AHA/NHLBI	0.90 (0.02)	0.97 (0.01)	0.94 (0.01)M
NCEP and IDF	0.53 (0.03)	0.80 (0.02)	0.70 (0.02)
AHA/NHLBI and IDF	0.55 (0.03)	0.82 (0.02)	0.71 (0.02)

### Prevalence of individual metabolic abnormalities

Using the NCEP definition of MetS, hypertension was the most frequent element in both sexes, followed by dyslipidemia (Table 5). The prevalence of central obesity was more than two times higher in women than in men (82.4 vs. 37.1%). Hyperglycemia was the least frequent MetS component in both men and women, regardless its definition.

**Table 5:** Sex-specific prevalence of individual metabolic abnormalities^1 ^among study participants with NCEP diagnosed metabolic syndrome

Metabolic abnormality	Prevalence					
	
	Men (N = 210)		Women (N = 347)		Total (N = 557)	
	
	%	95 % CI	%	95 % CI	%	95 % CI
Central obesity	37.1	30.6-43.7	82.4	78.4-86.5	65.4	61.4-69.3
High TG	83.8	78.8-88.8	71.2	66.4-76.0	76.7	73.1-80.2
Low HDL-C	85.7	80.9-90.5	88.8	85.4-92.1	87.6	84.9-90.4
AH	93.8	90.5-97.1	89.1	85.8-92.4	90.8	88.4-93.3
Elevated SG	33.3	26.9-39.8	18.4	14.3-22.5	24.1	20.5-27.6
Elevated HBA1c	17.1	12.0-22.3	14.4	10.7-18.1	15.4	12.4-18.5

^1 ^Central obesity: WC ≥88 cm (women) and ≥102 cm (men); High TG: triglycerides ≥1,7 mmol/l; Low HDL-C, high-density lipoprotein cholesterol <1,29 mmol/l (women) and <1,04 mmol/l (men); Arterial hypertension (AH): SBP ≥ 130 mmHg, or DBP ≥ 85 mmHg, or Rt for hypertension ; Elevated SG: serum glucose ≥6.1 mmol/l or self-reported DM, or Rt for hyperglycemia; Elevated HBA1c: glycated haemoglobin ≥6.1% or self-reported DM, or Rt for hyperglycemia

Almost three quarters of the men and more than two thirds of the women in the total study sample had at least one MetS component (Table 6). Altogether, 17.3% had three or more metabolic abnormalities, of these, 60.7% had three, 33.0% had four, and 6.3% had all five.

**Table 6:** Age-standardized^1 ^prevalence of one or more metabolic abnormalities among those who were diagnosed with metabolic syndrome according to NCEP definition

Number of abnormalities	Prevalence					
	
	Men (N = 1918)		Women (N = 1637)		Total (N = 3555)	
	
	%	95 % CI	%	95 % CI	%	95 % CI
≥1	72.5	70.7-74.3	68.7	66.7-70.7	70.8	69.5-72.2
≥2	31.4	29.3-33.5	39.5	37.4-41.6	35.4	33.9-36.9
≥3	12.4	10.9-14.0	22.4	20.5-24.3	17.3	16.0-18.5
≥4	4.0	3.0-4.9	9.9	8.4-11.3	6.8	5.9-7.7
5	0.55	0.2-0.9	1.7	1.0-2.3	1.1	0.7-1.5

^1 ^Standardized to the Russian population in 2002 by the following age-strata 20-29, 30-39, 40-49, 50-59, 60+ years.

## Discussion

To our knowledge, this is the first relatively large study addressing the prevalence of MetS and its components in Russia. While the prevalence of MetS among Russian women is comparable to the European and the USA data, the prevalence among Russian men is considerably lower than among their European and North-American counterparts. The low prevalence rates of MetS combined with the high cardiovascular mortality among Russian men need to be explored in further studies.

Assessment of the MetS' burden is the first step towards monitoring the occurrence of the syndrome and developing effective preventive measures for this condition in Russia. The use of different internationally accepted diagnostic criteria and different standardization procedures provide a unique opportunity for comparison with both international and Russian studies. However, the results should be interpreted with caution, taking into account several limitations of the study.

The method used to recruit the study population might to a certain degree have resulted in a residual "healthy worker effect". Unemployed and marginalized subsets of the population such as alcohol or drug abusers and the homeless were underrepresented. Exclusion of 150 individuals from the sample because of missing values might represent another weakness. However, the prevalence of individual metabolic components in this group did not differ significantly from the group included into analyses.

There was a potential for clinical-chemical measurement errors in the study. Glucose was measured in serum, not in plasma. Because serum has a higher content of water, the cut-off point for defining hyperglycemia should be slightly higher. Moreover, we assumed that all blood samples were fasting but this was not ensured. Thus, the estimate of the prevalence of MetS may be inaccurate. To address these methodological problems, we also calculated the prevalence of MetS using two alternative criteria for hyperglycemia. The first criterion involved accepting one of the following: HBA1c ≥ 6.1 %, or self-reported DM, or receiving treatment for high blood sugar. The HBA1c marker reflects the average level of glycemia over the preceding 2'3 months and does not depend on fasting. In this study, HBA1c was measured using a precise and reliable method certified by the US National Glycohemoglobin Standardization Program [27]. An earlier published meta-analysis [28] and a recent systematic literature review showed that the performance of HBA1c in detecting type 2 diabetes was comparable with that of fasting plasma glucose, and a cut-off point of ≥6.1 %. was recommended [29]. The MetS rates based on the HBA1c were the most conservative, since for the chosen cut-off point of ≥6.1 % the test identifies subjects with Impaired Fasting Glucose (IFG) with lower sensitivity than those with the diabetes. Thus, some participants having IFG were falsely labelled as having normoglycemia. The second criterion involved raising the cut-offs for serum glucose from 5.6 to 5.8 mmol/l (IDF and AHA/NHLBI), and from 6.1 to 6.3 mmol/l (NCEP), according to the local standards at the UNN laboratory. Agreement between these two *ad hoc *definitions of hyperglycemia was relatively fair (kappa = 0.68). However when comparing the MetS rates defined by the NCEP criteria based on these two *ad hoc *definitions of hyperglycemia, corresponding agreement was very good (kappa = 0.97). Similar results were seen for other definitions (IDF, AHA/NHLBI). This might be rationalized by the cluster nature of MetS and by the fact that hyperglycemia was the least prevalent metabolic abnormality in both genders. The impact of the latter on the probability of having MetS was minimal.

The new estimates obtained by applying these modified criteria were slightly lower than previously described (data not shown), but the agreements between all three estimates were ≥0.95, suggesting that the degree to which the prevalence of MetS was overestimated in this study is small.

Our findings on the prevalence of MetS among Russian women are comparable to corresponding studies from Europe [9-11] and the USA [7,8]. However while in these studies the prevalence among men was equal to or even higher than that among women, our study shows that among men it is almost half that among women. This remarkable disparity might be explained by lifestyle and socio-economic differences between the genders. Females were slightly older, better educated and primarily employed in jobs where the level of physical activity at work was low, e.g. school teachers, office workers, sewing-factory workers. There was a higher proportion of pensioners among the women than the men (23 vs. 15%, respectively). Men had higher levels of physical activity at work and consumed more alcohol, with vodka binge drinking as a prevailing pattern. Men also smoked substantially more. The prevalence rates of visceral adiposity (as defined by both the NCEP and IDF criteria), which is the core element in metabolic syndrome's pathogenesis and the proxy-indicator of insulin-resistance, was also much lower among men than women in our study. A detailed analysis of the relationships among MetS, its components, and life-style and socio-demographic determinants among Russian adults is beyond the scope of this paper.

Only one research publication was found that examined the prevalence of MetS among Russians [30]. It was carried out in the Kuzmolovsky district (close to St. Petersburg) and reported a much higher prevalence rate (54% vs. 18.9% in our study). However, that study had severe limitations: small sample size (146 participants), questionable selection procedure (i.e. shifted gender and age distribution; 91% were women with a mean age of 68 years), prevalence was reported without gender- and age-standardization, and a high prevalence of co-morbidity occurred (90% reported heart disease). Nevertheless, if we compare the results of that study with ours for women in the age group 60+, the difference in MetS' prevalence is reduced considerably (54% vs. 45%).

A population-based study of men living in Kuopio in Eastern Finland [31] reported a prevalence of MetS (using the NCEP definition) similar to ours (13.7% vs. 12.4% in our study). The population in that study was older (mean age 52 years vs. 41.6 years in our study), which might at least partly explain the higher prevalence in the Finnish sample. However, the Finnish study was performed in the late 1980s and more recent data from Finland suggest that the current prevalence of the MetS is higher. The results of a 2001 cross-sectional study in Slovakia [32] were comparable to ours, with a similar highly significant difference between men and women in the prevalence of NCEP-defined MetS (15.9% vs. 23.9%). However, there was no significant difference in IDF-defined MetS between genders in the Slovakian study. One may speculate about a specific distribution of the MetS by gender in Eastern European countries, but more data from this region are needed before definite conclusions are drawn.

The prevalence of MetS in the Arkhangelsk study increased progressively with age. This has also been observed in other studies, but to a lesser extent [7-11]. In our study, there was a fifteen-fold increase among women and a nine-fold increase among men when the 18-29 and 60+ age groups were compared. In the sub-sample of individuals with MetS, the most frequent metabolic abnormalities were arterial hypertension and low HDL-C. Only one-third of men diagnosed with MetS (NCEP) had central obesity, whereas more than 80% of women with MetS were obese. Interestingly, although the prevalence of central obesity was higher among women, the mean WC was higher among males in all age groups.

The method used to define obesity (BMI, WC or WHR) strikingly affected the reported prevalence of this condition in both genders. The variation was particularly striking among men, ranging from 6% to 26% using the WC and WHR definitions, respectively. By contrast, among women, the frequency was highest when obesity was defined by the WC-criteria. The original sex-specific thresholds for WC were originally (at least partly) established in cross-sectional studies from Holland and the UK [33,34], using correlation between WC and BMI in subjects with BMI ≥25 kg/m^2 ^and 30 kg/m^2^. The cut-offs for obesity using WC depend on ethnicity [25]. According to the ROC analysis, the optimal cut-offs for WCs corresponding to BMIs of ≥25 kg/m^2 ^and ≥30 kg/m^2 ^were about 10 cm lower (≥84 cm and ≥92 cm, respectively) than the original one (≥94 cm and ≥102 cm, respectively) suggesting a lower tendency for central adiposity at a given BMI among the men in our study setting. Similar results were reported from a study of middle-aged eastern Finnish men in the late 1980^th ^[31]. On the contrary, the standard cut-offs of WC for women (≥80 cm and ≥88 cm) originally corresponded well to BMIs of 25 kg/m^2 ^and 30 kg/m^2^. Therefore, the original cut-offs used for WC in NCEP and IDF definitions of MetS may be inappropriate for men living in Northwest Russia. This is an important finding that might largely explain the unequal distribution of MetS by sex. The finding needs further verification.

The most reasonable explanation for our main findings is the difference in life-style between Russian men and women: women smoked less, had lower alcohol consumption and, what is more important, lower levels of physical activity. In general, Russian women have occupations involving low levels of physical activity at work (service sector, office personnel, workers in the sewing industry), whereas men have higher levels of work-related physical activity.

Our study did not support the hypothesis that the high burden of cardiovascular morbidity and mortality among Russian men could be attributed to a high prevalence of MetS. A high prevalence of smoking and life-style associated with excessive alcohol consumption and specific drinking patterns may be other contributors to the high cardiovascular morbidity and mortality among Russian men. Alcohol consumption was high in our study population [35], especially among men. It might have had a "protective" effect on the development of the metabolic syndrome, but an opposite effect on the risk of fatal cardiovascular events. An alcohol-related increase in insulin sensitivity has been reported, involving a linearly-associated lower risk for MetS [36]. Nevertheless, alcohol consumption is an established risk factor for CVD mortality in Russia [37]. Analyses of associations between MetS and socio-demographic characteristics, smoking, alcohol and other factors, as well as cardiovascular risk attributable to MetS, are beyond the scope of this paper and will be pursued in future studies.

Differences in life expectancy between Russian men and women (59.0 and 72.3 years in 2000, respectively) may contribute to an explanation of the observed differences between genders in the prevalence of MetS [38], suggesting that Russian men do not reach the age when MetS becomes highly prevalent. Age-standardization, however, leveled out this difference only partially, suggesting that the difference in life expectancy is not the only contributor to the gender difference in the prevalence of MetS in Russia.

## Conclusion

While the prevalence of MetS among Russian women is comparable to the data for Europe and the USA, the prevalence among Russian men is considerably lower than among their European and North-American counterparts. Our results suggest that MetS is unlikely to be a major contributor to high mortality from cardiovascular diseases among Russian men. Further studies of determinants for MetS and its components, and MetS and cardiovascular risk are needed for a better understanding of the mechanisms leading to the exceptionally high cardiovascular mortality in Russia.

## Competing interests

The authors declare that they have no competing interests.

## Authors' contributions

OS, AMG and ON drafted the manuscript. ON, TB, VA and SM planned the study and collected the data. OS and TB analyzed the data. All authors read and approved the final version of the manuscript.

## Pre-publication history

The pre-publication history for this paper can be accessed here:

http://www.biomedcentral.com/1471-2458/10/23/prepub
